# Investigating the Potential of Poly(2-ethyl-2-oxazoline) and Its Polymer Blends for Enhancing Fenofibrate Amorphous Solid Dispersion Dissolution Profile

**DOI:** 10.3390/pharmaceutics17101238

**Published:** 2025-09-23

**Authors:** Ziru Zhang, Rasha M. Elkanayati, Sheng Feng, Indrajeet Karnik, Sateesh Kumar Vemula, Michael A. Repka

**Affiliations:** 1 Department of Pharmaceutics and Drug Delivery, School of Pharmacy, The University of Mississippi, Oxford, MS 38677, USA; zzhang5@go.olemiss.edu (Z.Z.); rmelkana@go.olemiss.edu (R.M.E.); sfeng1@go.olemiss.edu (S.F.); irkarnik@go.olemiss.edu (I.K.); 2 Department of Pharmaceutics, School of Pharmaceutical Sciences, Lovely Professional University, Phagwara 144411, India; 3 Pii Center for Pharmaceutical Technology, The University of Mississippi, Oxford, MS 38677, USA

**Keywords:** amorphous solid dispersions, fenofibrate, hot-melt extrusion, poly(2-ethyl-2-oxazoline), poorly water soluble, solubility enhancement

## Abstract

**Background/Objectives:** This study aimed to develop a novel amorphous solid dispersion (ASD) platform using poly(2-ethyl-2-oxazoline) (PEtOx) for the solubility enhancement of poorly water-soluble drugs. Fenofibrate (FB), a Biopharmaceutics Classification System (BCS) Class II drug, was selected as the model drug. The novelty of this work lies in the formulation of dual-matrix systems by blending PEtOx of varying molecular weights (50 kDa, 200 kDa, 500 kDa) with solubility-enhancing polymers, Soluplus^®^ and Kollidon^®^ VA64, to investigate component compatibility, synergistic solubility enhancement, and the influence of PEtOx molecular weight on drug release. **Methods**: ASDs were prepared via hot-melt extrusion (HME) and characterized using differential scanning calorimetry (DSC), scanning electron microscopy (SEM), powder X-ray diffraction (PXRD), and Fourier transform–infrared spectroscopy (FTIR) to confirm FB amorphization and evaluate drug–polymer interactions. In vitro dissolution testing was performed to assess drug release performance, and stability studies were conducted at ambient conditions for one month to evaluate physical stability. **Results:** DSC, PXRD, and FTIR confirmed the successful amorphization of FB and good miscibility between PEtOx and the selected excipients. In vitro dissolution studies showed an 8–12-fold increase in FB release from ASDs compared to crystalline drug. Lower-molecular-weight PEtOx grades yielded faster release profiles, while binary blends with Soluplus^®^ or Kollidon^®^ VA64 enabled tailored drug release. Stability testing indicated that all formulations maintained their amorphous state over one month. **Conclusions**: PEtOx-based ASDs represent a versatile platform for enhancing the solubility and dissolution of poorly water-soluble drugs. By adjusting polymer molecular weight and combining with complementary excipients, release profiles can be optimized to achieve improved performance and stability.

## 1. Introduction

For oral dosage forms, the bioavailability is critically influenced by several factors, including aqueous solubility, membrane permeability, dissolution rate, and susceptibility to first-pass metabolism [1,2,3]. Among these, poor aqueous solubility remains one of the most significant barriers to achieving adequate systemic drug exposure following oral administration. According to previously published statistics, it is estimated that over 70% of newly developed drug candidates in the market exhibit low water solubility [4,5]. To address this challenge, the pharmaceutical industry has developed various formulation strategies to enhance solubility and consequently oral bioavailability. These include the use of the cyclodextrin inclusion complex, nanoparticle formulations, co-crystals, colloidal drug delivery systems, and solid dispersions [5,6].

Amorphous solid dispersions (ASDs) improve the solubility and oral bioavailability of poorly water-soluble drugs, where the crystalline form is transformed into a high-energy amorphous form that is molecularly dispersed within a hydrophilic polymer matrix [7,8,9]. This approach eliminates the need to overcome the lattice energy required for dissolution in crystalline forms, resulting in enhanced dissolution rates and overall solubility. ASDs are broadly applicable to a wide range of active pharmaceutical ingredients (APIs), including acidic, basic, neutral, and zwitterionic drugs [10]. In addition, ASD shows several benefits over liquid and semi-solid formulations, such as lower manufacturing costs and compatibility with fixed-dose combinations [11]. Despite their benefits, amorphous systems are thermodynamically unstable and prone to recrystallization. Embedding the drug into a polymeric carrier helps mitigate this issue and enhances physical stability by increasing the glass transition temperature of the overall system, reducing molecular mobility, and enabling stabilizing interactions such as hydrogen bonds. Furthermore, the polymer can maintain drug supersaturation in the gastrointestinal tract, enhancing absorption and bioavailability.

Hot-melt extrusion (HME) is a widely adopted technique for the production of ASDs, particularly in the development of oral drug delivery systems. In this continuous process, the drug–polymer powder blend is continuously fed into the heated barrel and thoroughly mixed by the rotating screws inside the barrel [12]. The heat and shear stress help overcome the crystal lattice energy and convert the material into the amorphous state. Compared with other ASD preparation techniques, HME offers significant manufacturing advantages, as the molten drug–polymer mixture could be directly shaped into various solid forms, such as films, granules, and pellets for direct administration or downstream processing into a range of oral dosage forms, including tablets for immediate- or modified-release delivery [13]. HME is a simple and low-cost process that eliminates the challenges associated with selecting a common solvent for both hydrophobic drugs and hydrophilic polymers. The elimination of organic solvents simplifies regulatory compliance, improves safety, and enhances scalability, making HME one of the most common methods for ASD manufacturing [14,15]. 

Although a number of hydrophilic polymers have been successfully employed for ASD development via HME, such as Eudragit^®^ EPO, Soluplus^®^, polyvinylpyrrolidone-co-vinyl acetate 64 (Kollidon^®^ VA 64), and hydroxy propyl methylcellulose acetate succinate (HPMCAS) [16,17,18,19,20], the overall polymer selection remains limited. Furthermore, only a small fraction of these have translated into commercial products, underscoring the urgent need for novel polymeric carriers that are suitable for a broader range of APIs. 

Poly(2-alkyl-2-oxazolines) (PAOx) are promising candidates for use in drug delivery systems, due to their structural versatility and ease of processing. They are synthesized via cationic ring-opening polymerization (CROP) of 2-oxazolines, allowing precise control over molecular weight and side-chain structure. Functional groups can be introduced into the chain-end and side-chain, which leads to a wide range of solubility parameters [21]. Among them, poly(2-ethyl-2-oxazoline) (PEtOx) stands out for its complete water solubility at physiological temperatures (lower critical solution temperature ~60 °C) [22], high physical and chemical stability, and excellent biocompatibility. PEtOx has been approved by the FDA as a food additive [22,23,24,25]. These characteristics, combined with its synthetic versatility and stability, make PEtOx a promising matrix candidate for pharmaceutical applications, particularly in the development of amorphous solid dispersions (ASDs) [26,27]. 

Fenofibrate (FB) is a poorly water-soluble Biopharmaceutics Classification System (BCS) Class II prodrug used to lower triglycerides and LDL cholesterol. It is highly lipophilic (log *p* ≈ 5.2) and exhibits poor water solubility of about 100 µg/mL and low oral bioavailability of only about 30%, presenting a suitable model compound for evaluating solubility enhancement strategies [28,29,30]. 

Recent studies have demonstrated the potential of PEtOx as an effective carrier in ASDs, showing its ability to enhance the dissolution of poorly water-soluble drugs through HME. Likewise, solvent evaporation studies suggested that PEtOx could serve as a promising alternative to conventional polymers such as polyvinylpyrrolidone (PVP). However, despite these promising findings, existing research has primarily focused on evaluating PEtOx alone as an ASD matrix. The behavior of PEtOx when blended with other pharmaceutical polymers remains largely unexplored. This strategy may exploit complementary properties to further improve solubility and drug release. 

To address this gap, the present study systematically investigates the use of PEtOx of varying molecular weights (50, 200, and 500 kDa) in combination with Soluplus (a hydrophilic polymer) and PVP VA 64 (an amphiphilic polymer) to formulate FB-loaded ASDs using HME. Soluplus and PVP VA 64 were selected since they are amphiphilic carriers with both hydrophilic and hydrophobic domains. This amphiphilic nature enables them to interact favorably with lipophilic drug molecules, improving miscibility, maintaining the amorphous state, and facilitating drug release. In addition, both polymers are pharmaceutically well-established excipients with prior evidence of effectiveness in stabilizing ASDs of poorly water-soluble compounds. The formulations were prepared at a predefined ratio. The study further identifies the optimum extrusion temperature, and evaluates the influence of PEtOx molecular weight and blend composition on the dissolution performance. Ultimately, this work aims to optimize formulation strategies and determine whether PEtOx-based dual polymer blends can provide synergistic benefits for improving the solubility of poorly water-soluble drugs. 

## 2. Materials and Methods

### 2.1. Materials

FB was kindly donated by Ashland Inc. (Lexington, KY, USA). PEtOx with molecular weights of 50, 200, and 500 kDa was donated by Polymer Chemistry Innovations, Inc. (Tucson, AZ, USA). Soluplus and Kollidon VA 64 were kindly provided by BASF (Ludwigshafen, Germany). The gelatin capsules (00 size) were purchased from Total Pharmacy Supply (Arlington, TX, USA). Sodium lauryl sulfate (SLS) was purchased from Spectrum Chemical (New Brunswick, NJ, USA). All other chemicals used were of analytical grade.

### 2.2. TGA

Thermogravimetric analysis (TGA) was conducted to evaluate the thermal stability of the individual components by monitoring weight loss as a function of temperature. Approximately 10–20 mg of pure FB, PEtOx, soluplus, and PVP-VA64 samples were placed in open high-temperature platinum pans and analyzed using a thermogravimetric analyzer, TGA 55 (TA Instruments, New Castle, DE, USA). The samples were heated from 25 °C to 500 °C at a heating rate of 10 °C/min under a nitrogen atmosphere, with a purge flow of 60 mL/min for the sample and 40 mL/min for the balance. Data acquisition and interpretation were carried out using Trios software (version 2.11.0.729).

### 2.3. Preparation of Physical Mixtures (PMs) and the ASDs Using HME

The composition of the PMs used for the HME process is shown in Table 1. Formulations F1 through F3 consisted of 30% drug load and 70% of PEtOx of varying molecular weights (50, 200, and 500 kDa), respectively, to assess the impact of polymer chain length on formulation properties. Formulations F4 and F5 utilized a binary polymer blend composed of PEtOx (50 kDa) with either Soluplus or PVP-VA 64 in a 1:1 ratio, while formulations F6 and F7 employed the same binary blend but substituted PEtOx with a higher-molecular-weight grade (500 kDa).

PEtOx, supplied as light yellow small pellets, was first milled using a coffee blender to ensure uniform distribution of the polymer within the PMs. Soluplus and PVP-VA 64, both received as fine white powder, were directly blended without preprocessing. The milled PEtOx and pure FB were sieved (#30 mesh sieve) to ensure uniform particle size and tumble-mixed at 25 rpm for 20 min in a V-shaped blender (MaxiBlend™, GlobePharma, Jersey city, NJ, USA). 

The prepared PMs were processed using a conical (5–14 mm), co-rotating twin-screw HAAKE™ MiniLab II Micro-Compounder Extruder (Thermo Scientific™, Karlsruhe, Germany). The material was manually fed into the extruder, and the feed rate was kept constant, with a screw speed of 30 rpm. The extrusion temperatures ranged from 100 °C to 125 °C, selected initially based on the melting point (T_m_) of the API and the glass transition temperature (T_g_) of the polymer(s) and further optimized according to the processability and quality of the extrudates. Following extrusion, the extrudates were cooled to room temperature and subsequently milled using a laboratory mixer. The milled ASDs were passed through a #30 ASTM mesh sieve and stored in sealed containers for further use.

### 2.4. Differential Scanning Calorimetry (DSC) and Modulated-DSC 

Crystallinity as a function of heat flow was investigated for the pure ingredients and milled ASDs using a differential scanning calorimetry (DSC) system (TA Instruments, New Castle, DE, USA). Following weighing (3–5 mg), samples were hermetically sealed in aluminum pans. The pans were loaded into the DSC chamber opposite a reference pan and subjected to a heating ramp from 25 °C to 150 °C at 10 °C/min, with nitrogen purge gas maintained at 50 mL/min. The resulting data and thermograms were processed using the Trios software (version 2.11.0.729).

Modulated DSC (M-DSC) was employed to accurately determine the glass transition temperature (T_g_) of the individual polymers and the developed solid dispersion. Approximately 5 mg of each sample was equilibrated at 0 °C, followed by heating from 0 °C to 200 °C at a rate of 5 °C/min, with a modulation of 1 °C every 60 s. Tg was determined from the midpoint of the reversing heat-flow signal.

### 2.5. Powder X-Ray Diffraction Analysis (PXRD) 

The crystallinity of the pure ingredients and ASDs was determined via a Rigaku X-ray system (D/MAX-2500PC, Rigaku Corp., Tokyo, Japan). Cu Kα radiation (λ = 1.54056 Å) was employed, with the X-ray tube operated at 40 kV and 15 mA. Scans were performed over the angular range of 5° to 40° (2θ) in step-scan mode. A step width of 0.01° and a scan rate of 10.00°/min were used.

### 2.6. Scanning Electron Microscope (SEM) 

The surface morphology of the PM and the extruded ASD was examined with a JSM-7200 FLV Scanning Electron Microscope (JEOL, Peabody, MA, USA) operated at an accelerating voltage of 5 kV. All samples were mounted on SEM stubs and secured with double-adhesive tape. Prior to imaging, the samples were sputter-coated with platinum under an argon atmosphere using a fully automated Denton Desk V TSC Sputter Coater (Denton Vacuum, Moorestown, NJ, USA).

### 2.7. Fourier Transform Infrared Spectroscopy (FTIR) 

Drug–excipient compatibility in the ASDs was evaluated using a Cary630 FTIR spectrometer (Agilent Technologies, Santa Clara, CA, USA), and the sample analysis was performed with a MIRacle Attenuated Total Reflection accessory (Pike Technologies, Madison, WI, USA) equipped with a single-bounce, diamond-coated zinc selenide (ZnSe) internal reflection element. The IR Spectra were collected over the wavenumber range 650–4000 cm^−1^ at a resolution of 4 cm^−1^. Samples (~10 mg), including pure FB, pure PEtOxs, physical mixtures (PMs), and ASDs, were positioned on the crystal surface and compressed using the integrated pressure tower to ensure uniform solid–crystal contact.

### 2.8. In Vitro Dissolution Study

For the dissolution study, 200 mg of the ASDs and pure FB were manually fed into 00 size hard gelatin capsules, and the dissolution studies were conducted using a United States Pharmacopeia (USP) II dissolution apparatus (Hanson SR8-plusTM; Hanson Research, USA) at a temperature of 37 ± 0.5 °C. The speed was set to 50 rpm. The dissolution media consisted of 900 mL of distilled water with 0.05 M SLS, as recommended by the USP guidance [31]. At predetermined time intervals, two mL samples were collected, filtered through a 0.45 mm membrane filter (Durapore^®^; Millipore Sigma, St. Louis, MA, USA), and appropriately diluted. An equal volume of fresh, pre-warmed dissolution medium was immediately added to replace the withdrawn samples. The drug concentration in each sample was analyzed using a UV-Vis spectrophotometer (Genesys 180, Thermo Scientific), at a wavelength of 288 nm.

The cumulative percentage of drug released was plotted against time. The amount of drug released at 15 min (Q15) was determined. The initial dissolution rate (IDR) was calculated as the percentage of drug dissolved within the first 15 min, divided by time (min^−1^). Dissolution efficiency (DE) was assessed by calculating the area under the dissolution curve up to a specific time point using the trapezoidal method and was expressed as a percentage of the area of a theoretical rectangle representing 100% drug release over the same duration. The relative dissolution rate (RDR) was obtained by comparing the amount of drug released from the optimized formulation with that of the conventional formulation at 15 min [32]. 

The similarity factor (*f*_2_) for drug release was calculated to compare between the formulations using the following equation, where an *f*_2_ value >50 indicates similarity between the initial and stored sample.

(1) $$f_{2}=50\times log\left\{{\left[1+\left(1∕n\right)\sum_{j=1}^{n}{\left|R_{j}-T_{j}\right|}^{2}\right]}^{-0.5}\times 100\right\}$$

where $f_{2}$ = similarity factor used in comparing two dissolution profiles; 

Rt = cumulative drug release of initial samples;

Tt = cumulative release of the test sample at predetermined time points;

N = number of time points.

### 2.9. Statistical Analysis 

The statistical analysis of the in vitro drug release data was performed using SPSS software (IBM Corp. Released 2010. IBM SPSS Statistics for Windows, Version 19.0. Armonk, NY, USA: IBMCorp.). Comparisons between the release of ASDs were performed using the unpaired *t*-test, with a significance level set at *p* < 0.05.

### 2.10. Storage Stability Study

The milled ASDs were stored in a sealed container with 1 g of desiccant inside. The sealed formulations were stored at room temperature for 1 month. To assess the stability, drug content, and DSC, profiles of the stored samples were compared with the freshly prepared samples.

## 3. Results

### 3.1. TGA 

**Figure 1:**
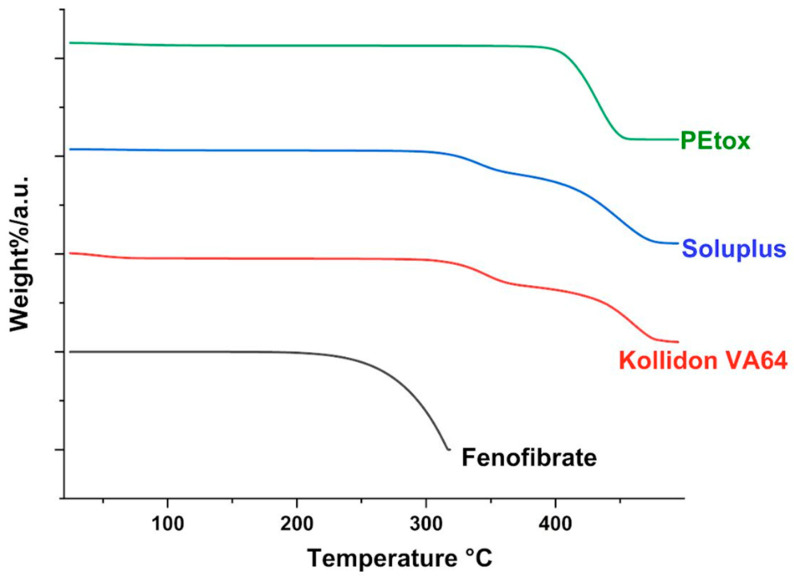
TGA thermogram of the drug and the pure excipients.

Before performing the extrusion process, it is necessary to identify the drug and the excipients’ degradation temperatures. The extrusion temperature should always be under the degradation temperature of all ingredients to avoid any drug loss or potential interaction. TGA was conducted to determine the temperature-dependent weight loss profiles of FB, PEtOx, Soluplus, and VA64. As shown in Figure 1, a minor weight loss observed below 100 °C was attributed to the evaporation of residual moisture, which is typical for hygroscopic materials. Significant weight loss at higher temperatures indicated the onset of thermal degradation. FB showed weight loss around 220 °C, while all three polymers, PEtOx, solulpus, and PVP-VA 64, demonstrated excellent thermal stability, with major decomposition occurring above 300 °C. For all excipients, there is negligible degradation under 200 °C, indicating an extrusion temperature under 200 °C is appropriate for ASD preparation.

### 3.2. Hot-Melt Extrusion 

**Figure 2:**
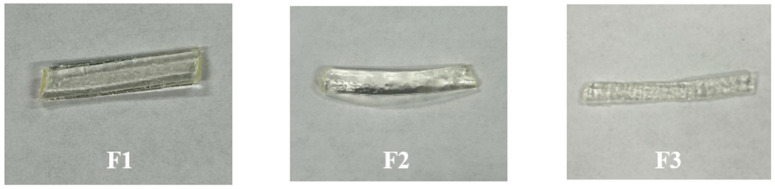
Different extrudates of formulations F1, F2 and F3 at an extrusion temperature of 100–105 °C.

The selection of an appropriate extrusion temperature is critical, as it significantly influences the physical characteristics of the extrudate and the successful formation of the ASD [33]. As shown in Figure 2, formulations F1 and F2, containing lower-molecular-weight PEtOx, were successfully extruded with a smooth surface at the temperature of 100 °C and 105 °C, respectively. However, at this temperature range, F3 extrudates, formulated with 500 kDa PEtOx, showed a rough surface with undissolved particles at these lower temperatures, likely due to incomplete mixing of the drug and polymer powder. This was also associated with increased torque during processing, indicating higher resistance within the extruder. However, when the temperature was raised to 125 °C, the roughness disappeared and the appearance of the F3 extrudate was similar to other formulations. A comparable trend was observed in the binary polymer systems where formulations incorporating 50 kDa PEtOx (F4 and F5) extruded well at 100 °C, while those containing 500 kDa PEtOx (F6 and F7) required a higher temperature (120 °C) to achieve complete dissolution and uniformity. Thus, as the polymer molecular weight increases, a corresponding elevation in extrusion temperature is necessary to ensure full dissolution of the components. 

Furthermore, this highlights the importance of temperature optimization where lower temperatures may lead to incomplete drug amorphization, high torque values, or rough extrudates due to undissolved components. Conversely, temperatures that exceed the necessary threshold can soften the extrudate excessively, resulting in filament that is overly soft or semi-liquid, hindering solidification and subsequent milling. Therefore, careful balancing of temperature is essential to ensure process efficiency and product quality. 

Notably, we observed that the extrusion temperature remained unchanged when PEtOx 50 (F1) was blended with either Soluplus (F4) or VA 64 (F5), compared to pure PEtOx 50 (F1). In contrast, the extrusion temperature decreased when PEtOx 500 was blended with Soluplus (F6) or VA 64 (F7), relative to pure PEtOx 500 ASD (F3). This phenomenon can be attributed to the molecular weight proximity between PEtOx 50 (~50,000 g/mol) and the other polymers (Soluplus: ~90,000–140,000 g/mol; VA 64: ~45,000–70,000 g/mol). Their comparable molecular weights result in a blend system where the average molecular weight is not significantly altered. However, blending the much higher molecular weight PEtOx 500 (~500,000 g/mol) with either Soluplus or VA 64 substantially reduces the average molecular weight of the mixture, resulting in extrusion at a lower temperature.

### 3.3. Thermal Analysis with DSC

**Figure 3:**
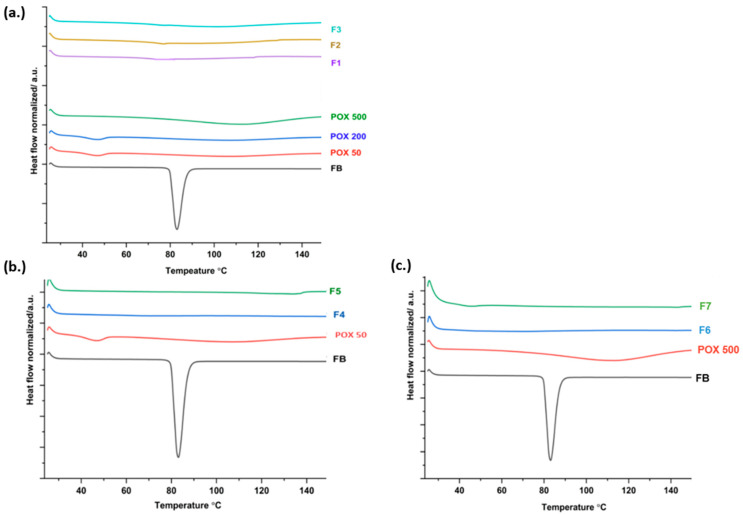
DSC thermogram of the pure ingredients compared to (**a**) F1, F2 and F3 (**b**) F4 and F5 (**c**) F6 and F7.

The thermal behavior of the pure API, PEtOx, and the corresponding physical mixtures and extrudates was evaluated using DSC to assess the crystallinity of the produced ASDs. The homogeneous distribution of the drug within the polymer matrix is an important indicator of a successful ASD. Achieving molecular-level dispersion of the API in the polymer can lead to ideal release of the API and contribute to improved physical stability during storage [34]. As shown in Figure 3, the DSC thermogram of crystalline FB exhibited a sharp endothermic peak at approximately 83 °C, with an enthalpy of fusion of 82.7 J/g, corresponding to its melting point. PEtOx, in contrast, is an amorphous polymer characterized by a glass transition temperature (T_g_), around 60 °C [35]. The disappearance of the drug’s melting peak in the extrudates thermograms confirms the absence of residual crystallinity and suggests successful amorphization and homogeneous dispersion of FB within the polymer matrix. As shown in Figure S1, the DSC thermograms of the physical mixtures exhibit clear melting endotherms, whereas these transitions are absent in the corresponding solid dispersions, thereby confirming this conclusion.

MDSC was conducted to identify the glass transition temperatures (T_g_s) of the bulk polymers, and it was estimated at about 58.7 °C, 67.7 °C, and 105 °C for Soluplus, PEtOx 50, and PVP VA, respectively. For the ASDs, F1 displayed a single T_g_ at ~33 °C, significantly lower than that of the polymers, which could be due to a plasticizer effect of the drug. This may lead to reduced kinetic stability and a tendency toward recrystallization during storage. By contrast, F4 showed two distinct T_g_s at about 63 °C and 100 °C, indicative of partial miscibility and the coexistence of two amorphous phases. The presence of two transitions suggests incomplete molecular dispersion of the drug.

F5 demonstrated a single T_g_ at about 118 °C. The presence of one T_g_ confirms the formation of a homogeneous amorphous phase with the drug molecularly dispersed within the polymer matrix. The high T_g_ should reduce molecular mobility, thereby improving the stability and minimizing the recrystallization tendency. M-DSC thermograms of selected extrudates (F4, and F5) are displayed in (Figure S2). 

Taken together, these results demonstrate that while F1 promotes rapid release, its low T_g_ could compromise the stability. F4 provides partial stabilization, but incomplete miscibility remains. F5 achieves molecular dispersion of the drug with a high T_g_ and enhanced resistance to recrystallization.

### 3.4. PXRD Analysis

**Figure 4:**
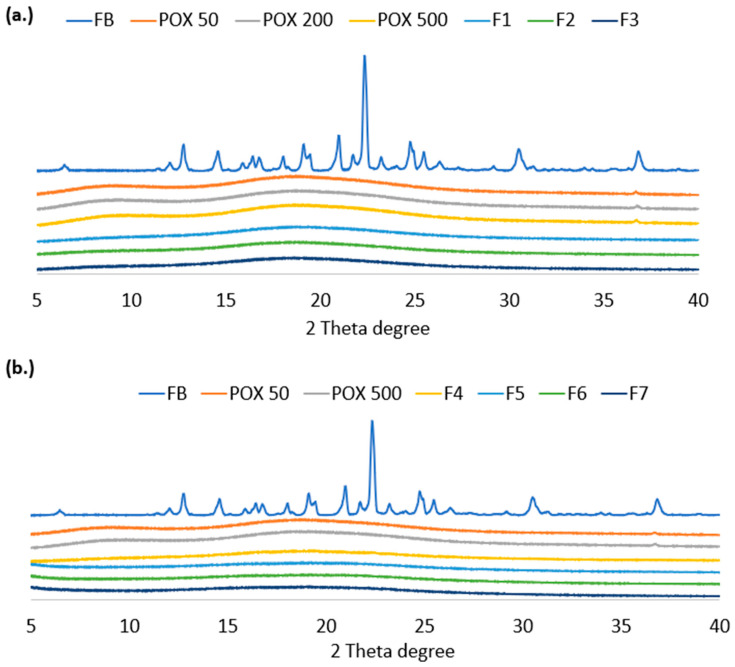
PXRD of the pure excipients compared to (**a**) F1, F2 and F3 ASDs, and (**b**) F4, F5, F6 and F7.

PXRD analysis was performed to confirm the amorphous state of FB in the extruded formulations and to support the DSC findings. As illustrated in Figure 4, the diffractograms demonstrate the complete disappearance of FB characteristic crystalline peaks in the formulations, relative to the distinct diffraction patterns of crystalline FB and the amorphous halos of the individual polymers (PEtOx/Soluplus/VA 64). Crystalline FB exhibited sharp peaks at the diffraction angles of 12.71°, 14.57°, 19.43°, 20.85°, 22.32°, 24.77°, and 36.78°, indicative of its long-range molecular order. In contrast, these peaks were entirely absent in all extrudates. These observations verify successful crystalline-to-amorphous phase transformation of FB during extrusion, and the effective dispersion of the drug within the polymer matrix. Notably, PEtOx appears to inhibit FB aggregation, preventing crystal nucleation [36].

### 3.5. SEM Analysis 

**Figure 5:**
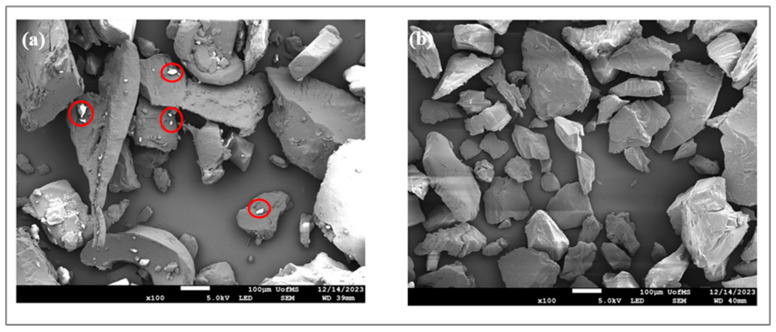
SEM of F3 PM and the extrudate. (**a**) PM; (**b**) extrudates.

The SEM study was performed to investigate the surface morphology of the PM and the extrudates. As shown in Figure 5a, the PM exhibited several small crystals surrounding irregularly shaped polymer particles, indicating a heterogeneous composition, and insufficient entrapment of FB within the polymer carriers. In contrast, Figure 5b, at the same magnification power, revealed a smooth and uniform surface with no visible FB crystals in the ASD. The SEM observations are consistent with the PXRD and DSC results, collectively confirming the successful transition of FB into the amorphous state and its uniform incorporation within the extruded matrix.

### 3.6. FTIR Analysis 

**Figure 6:**
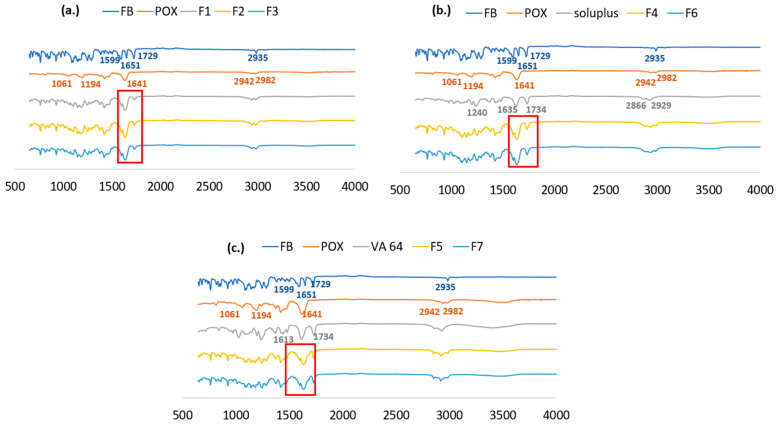
FTIR of the pure excipients and (**a**) F1, F2 and F3; (**b**) F4 and F6; (**c**) F5 and F7. Red circled parts indicate 1650–1750 cm^−1^ (carbonyl stretching) where band broadening and loss splitting are observed.

FTIR was conducted to investigate the potential interactions between the API and the polymers [37], which may influence ASD stability. Also, minimizing the intermolecular force between the API and polymers would greatly contribute to drug dissolution [37,38]. As shown in Figure 6, the pure FB shows characteristic peaks at ~1599 cm ^− 1^ for the C=O ketone group, ~1729 cm^−1^ for the C=O stretching of the ester group, and ~2935 cm^−1^ for the benzene ring [39,40].

In the FTIR spectrum of PEtOx, characteristic absorption bands are observed at 1061 cm^−1^ and 1194 cm^−1^, assigned to C-C stretching vibrations. Peaks within the 1200–1480 cm^−1^ range are associated with C-H group deformations. The distinct band at 1641 cm^−1^ is attributed to carbonyl (C=O) vibrational modes, while the higher-frequency bands at 2942 cm^−1^ and 2982 cm^−1^ originate from asymmetric stretching vibrations of methyl (-CH_3_) groups [41,42]. For soluplus, the FTIR spectrum exhibited characteristic absorption bands at 2929/2866 cm^−1^ (C-H stretching), 1734 cm^−1^ (ester C=O), 1635 cm^−1^ (tertiary amide C=O), and 1240 cm^−1^ (ester C-O), confirming the expected functional groups [43]. As for VA64, the FTIR spectra display two characteristic stretching vibrations, including a peak near 1734 cm^−1^ attributed to the vinyl acetate monomer, and another at approximately 1613 cm^−1^ corresponding to the vinyl pyrrolidone monomer [44]. 

For the extruded formulations, a shift of fenofibrate from a crystalline to an amorphous state was observed. Generally, infrared spectral analysis reveals distinct band broadening, loss of splitting and peak coalescence in amorphous fenofibrate relative to its crystalline form, attributable to reduced structural uniformity, and increased molecular conformation variability with heterogeneous intermolecular interactions [45]. This phenomenon is particularly evident in the 1650–1750 cm^−1^ (carbonyl stretching) and 2900–3100 cm^−1^ (C-H stretching) regions, where spectral resolution decreases through peak merging and bandwidth expansion, as demonstrated in Figure 6. In the crystalline state, the highly ordered lattice restricts functional groups to nearly identical molecular environments, producing sharp spectral peaks. In contrast, the absence of long-range order in the amorphous form leads to a distribution of local molecular environments, with variations in bond lengths, angles, and intermolecular interactions. This structural heterogeneity is responsible for the significant broadening of the absorption bands [39,46].

Loss of splitting of the peak can also be observed in these regions. Crystalline fenofibrate exhibits split peaks or shoulders due to vibrational coupling or crystal field effects. These features disappear in the amorphous state, resulting in smoother, more featureless peaks. This is because the loss of long-range order and molecular symmetry in the amorphous state eliminates vibrational coupling and crystal field effects that cause splitting in crystalline spectra [39,47].

Notably, characteristic carbonyl stretching vibrations exhibit measurable shifts from 1651/1729 cm^−1^ in the crystalline phase to 1636/1733 cm^−1^ in the amorphous state corresponding to Δν ≈ −15 cm^−1^ for the ketone C=O and Δν ≈ +4 cm^−1^ for the ester C=O. The ketone carbonyl maximum shifts from ~1651 cm^−1^ in crystalline fenofibrate to ~1632–1636 cm^−1^ in the dispersions (Δ ≈ −15 cm^−1^), which accompanies loss of crystal packing. The shifts may indicate some degree of interaction between the amorphous FB and PEtOx.

These spectral modifications are consistent with the crystalline-to-amorphous transition patterns documented by Heinz et al., with the generated FTIR data conclusively serving as an indicator of amorphization, which is in agreement with PXRD and DSC results [39,41,42,43,44,45].

### 3.7. In Vitro Dissolution 

**Figure 7:**
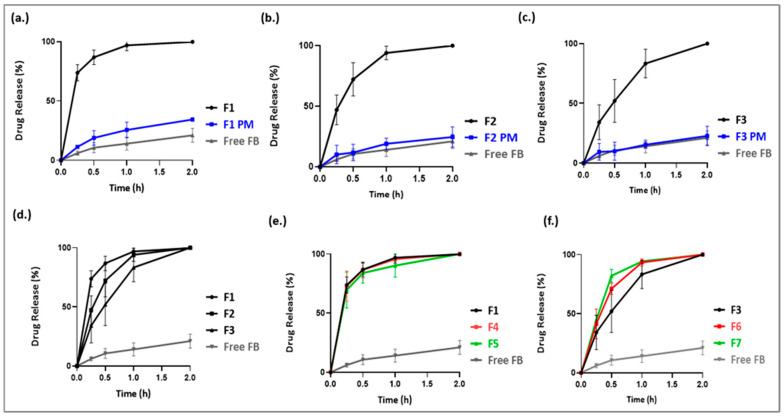
In vitro dissolution profile of the pure drug and (**a**) F1 PM and extrudate; (**b**) F2 PM and extrudate; (**c**) F3 PM and extrudate; (**d**) F1, F2 and F3 extrudates; (**e**) F1, F4 and F5 extrudates; (**f**) F3, F6 and F7 extrudates (*n* = 3).

The dissolution profiles shown in Figure 7 indicate that free FB exhibited poor performance, with only 20% of the drug dissolved within two hours. In contrast, formulations F1 and F2 achieved complete drug release (100%) within one hour, while F3 reached 100% release within two hours, demonstrating a five-fold enhancement in release profiles compared to the free drug. Notably, F1 displayed the most rapid and significant improvement, releasing over 87% of the drug within 0.5 h, representing an eight-fold increase relative to the free drug. Conversely, F3 showed the slowest release profile among the three formulations, suggesting that lower-molecular-weight PEtOx is more effective in enhancing the rate and extent of drug release.

The corresponding PMs of F1–F3 exhibited drug releases of 34%, 25%, and 23%, respectively, within the same timeframe. The slight improvement in PM release compared to the free drug may be attributed to the inherent solubilizing properties of hydrophilic PEtOx; however, this effect was negligible compared to the fabricated PEtOx-based ASDs (F1–F3), which further confirms that drug amorphization played a pivotal role in enhancing dissolution. Amorphous drugs dissolve more readily due to the lack of a crystalline lattice, which reduces the energy required for dissolution. Their disordered molecular structure also enables greater molecular mobility and promotes interactions such as hydrogen bonding with the dissolution medium. This structural randomness allows solvent molecules to more easily access and dissolve the drug. These findings are consistent with DSC and PXRD data, which support the successful amorphization of the API. 

Formulations F4 and F5, prepared by blending PEtOx50 with Soluplus or VA64 at a 1:1 ratio (Figure 7e), exhibited dissolution profiles comparable to F1. This indicates that the addition of Soluplus or VA64 did not further enhance drug release, and that the dissolution improvement can be primarily attributed to PEtOx50. 

However, blending PEtOx500 with either Soluplus or VA64 (Figure 7f) resulted in a significant improvement in drug dissolution profile (*p* < 0.05), emphasizing that combining polymers of different molecular weights or functionality can be an effective strategy for tailoring release profiles to align with specific therapeutic goals. Among the binary systems, the PEtOx500–PVP VA64 blend exhibited a slightly faster release compared to the Soluplus blend. Furthermore, the results underscore the superior solubility enhancement potential of PEtOx50, which demonstrated the most rapid release overall, highlighting the importance of polymer molecular weight in modulating release kinetics. 

Dissolution studies showed that formulations F1, F4, F5, and F7 successfully met the USP dissolution criteria for immediate-release oral dosage forms, at over 80% within 30 min [31]. These results further underscore the superior solubility-enhancing capability of PEtOx 50, evident both in its pure form (F1) and within binary blends (F4, F5). Notably, the performance of F7 demonstrates that while PEtOx 500 exhibits relatively inferior solubility enhancement when used as a single-component matrix compared to PEtOx 50, it can effectively synergize with another polymer (VA 64 in this case) within a dual-matrix system to achieve robust solubility enhancement and meet critical dissolution requirements.

Table 2 explains the superiority of ASDs when compared to free drug in terms of Q15, IDR, and DE. The dissolution study revealed a significant enhancement in drug release from the ASD formulations compared to the free drug, particularly in early-phase performance. Formulations F1 and F4 showed the highest drug release at 15 min (Q15 > 72%), along with rapid initial dissolution rates (IDR > 4.8%/min), indicating their strong potential for immediate therapeutic action. These improvements are likely due to formulation strategies such as amorphous solid dispersion and incorporation of hydrophilic excipients that enhance wetting and disintegration. The relative dissolution rate, RDR, further emphasized this effect, with over 11–fold improvement in early release compared to the free drug. Dissolution efficiency, a more comprehensive metric, confirmed the overall superiority of these formulations [48] DE, which accounts for the overall drug release behavior over time, was highest for F1 (75.18%) and F4 (74.51%), followed by F5 (71.38%). These values were substantially greater than those of the free drug (9.00%), reinforcing the superior dissolution profiles of the optimized formulations. Formulations F6 and F7, showed moderate improvements (DE values of 60.36% and 65.53%, respectively). The similarity factor (f_2_) for the relevant comparisons was calculated in accordance with FDA/EMA guidance. The results show that F1 vs. F4 yielded f_2_ = 93, and F1 vs. F5 yielded f_2_ = 71, indicating similar dissolution profiles to F1.

In contrast, F1 vs. F6 (f_2_ = 34) and F1 vs. F7 (f_2_ = 39) were not similar, reflecting slower early-time dissolution with the high-MW PEtOx blends. These findings confirm that amphiphilic blends with PEtOx-50 (F4/F5) maintain F1-like release while offering improved physical stability, whereas blends with PEtOx-500 (F6/F7) demonstrated slower release before 60 min.

### 3.8. Stability Study

**Figure 8:**
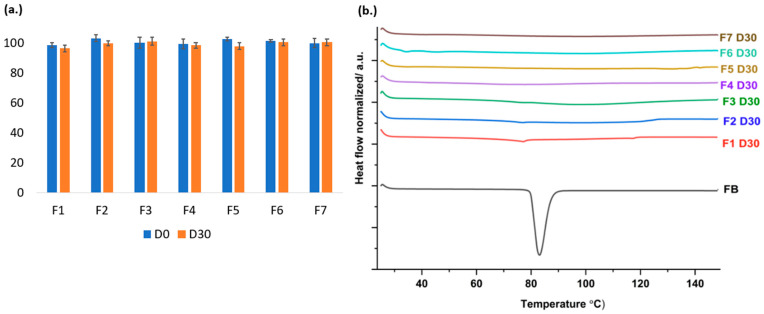
Storage stability results. (**a**) Drug content; (**b**) DSC (*n* = 3).

ASDs with solubility-enhancing polymers and high drug loading are inherently prone to recrystallization, necessitating stability assessment for appropriate storage guidance [49]. As shown in Figure 8, the drug content of the fresh formulation was between 98.53% and 103.12%. The drug content after one-month storage remained within an acceptable range of 96.38–101.12%, with minimal deviation from initial values. DSC thermograms revealed the appearance of a small endothermic peak in formulations F1 and F2 after 1 month, suggesting the onset of partial recrystallization. In contrast, no recrystallization was observed in formulations F4, F5, F6, and F7, which maintained their amorphous nature. These findings suggest that incorporating high-molecular-weight PEtOx or polymer blends plays a critical role in balancing drug release performance and physical stability, with higher-molecular-weight systems offering enhanced resistance to recrystallization during storage. As shown in Figures S3 and S4, F1 PM exhibits the lowest enthalpy of mixing (26.03 J/g) at 81 °C, reflecting higher drug–polymer miscibility and rapid dissolution. However, partial recrystallization occurred after one month, likely due to the low molecular weight (~50 kDa) and low T_g_ (~67.7 °C) of PEtOx 50, which increase free volume and chain mobility, facilitating molecular diffusion and nucleation [50,51,52]. This aligns with the F1 DSC result. Thus, although F1 shows excellent dissolution, it suffers from poor kinetic stability.

In contrast, blending PEtOx 50 with higher-MW polymers such as Soluplus^®^ (~90–140 kDa) or PVP-VA64 (~45–70 kDa) significantly improved physical stability. As shown in (Figure S4), the physical mixtures of F4 (33.8 J/g) and F5 (30.58 J/g) displayed higher enthalpies relative to F1, reflecting slightly reduced miscibility but substantially suppressed recrystallization. The higher molecular weight enhances topological constraints and reduces segmental mobility. In summary, while F1 offers optimal dissolution, binary blends leverage the high-MW carrier properties, achieving a more favorable compromise between dissolution performance and long-term stability.

## 4. Conclusions

Different grades of polymer-based formulations were successfully prepared using HME technology. Characterization via DSC, PXRD, FTIR, and SEM confirmed the amorphous nature of all formulations post extrusion, with no detectable crystallinity. The ASDs containing PEtOx combined with Soluplus or VA64 demonstrated excellent miscibility, as evidenced by homogeneous phase behavior in DSC studies. Dissolution studies revealed an 8-to 12-fold improvement in drug release profiles compared to the free drug, with all formulations achieving complete drug release within 2 h. Among these, PEtOx-50 exhibited the most pronounced solubility improvement, attributed to its optimal molecular weight. Storage stability studies indicated that the ASDs remained physically stable for at least one month in sealed containers, with no recrystallization observed during this period. Generally, these results highlight PEtOx as a promising matrix for ASDs, capable of enhancing the solubility and, consequently, the bioavailability of poorly water-soluble drugs. Furthermore, PEtOx demonstrated good compatibility when blended with other solubility-enhancing polymers such as Soluplus and VA64, suggesting its versatility in multi-polymer systems. The release kinetics of PEtOx-based ASDs can be tailored by blending higher-molecular-weight grades of the polymer with lower-molecular-weight hydrophilic polymers, offering more rapid drug release. Further studies should focus on optimizing polymer ratios in the binary system to balance dissolution and stability, along with investigating the influence on milling particle size of the ASDs and developing an in vitro–in vivo correlation (IVIVC).

## Figures and Tables

**Table 1:** Composition and extrusion information of each formulation.

Formulations	FB	PEtOx	PEtOx	Soluplus	PVP-VA 64	Extrusion Temperature	Torque
	(%)	Mwt (KDa)	%	%	%	(°C)	(N·cm)
F1	30	50	70	-	-	100	11–12
F2	30	200	70	-	-	105	12–13
F3	30	500	70	-	-	125	12–15
F4	30	50	35	35	-	100	10–12
F5	30	50	35	-	35	100	13–15
F6	30	500	35	35	-	120	13–15
F7	30	500	35	-	35	115	9–11

**Table 2:** Comparison of in vitro dissolution parameters of ASDs with free drug.

Dissolution Parameters	F1	F4	F5	F6	F7	Free Drug
Q15	73.74	72.82	69.55	41.37	44.90	6.08
IDR	4.92	4.85	4.64	2.76	2.99	0.41
RDR	12.14	11.99	11.45	6.81	7.39	Reference
DE	75.18	74.51	71.38	60.36	65.53	9.00

## Data Availability

The data presented in this study are available within the article.

## Supplementary Materials

The following supporting information can be downloaded at: https://www.mdpi.com/article/10.3390/pharmaceutics17101238/s1, Figure S1: DSC of FB, selected ASDs and corresponding PMs; Figure S2: (A) M-DSC for F4 extrudate, and (B) F5 extrudate displaying a molecular dispersion with Tg of about 118 °C; Figure S3: DSC of F1 PM demonstrating a melting endotherm of about 26 J/g; Figure S4: (A) DSC of PM1, PM4 and PM5 (B) Overlay of the physical mixtures showing lowest enthalpy of PM1 compared to PM4, and PM5.
