# Supplementary material for: Investigating the Potential of Poly(2-ethyl-2-oxazoline) and Its Polymer Blends for Enhancing Fenofibrate Amorphous Solid Dispersion Dissolution Profile
Source: Pharmaceutics. 2025 Sep 23;17(10):1238. doi: 10.3390/pharmaceutics17101238 (PMC12566808; doi:10.3390/pharmaceutics17101238)
Supplement: Supplementary file 1 [file pharmaceutics-17-01238-s001.zip › pharmaceutics-3844009-supplementary.pdf]

pharmaceutics

Article

Investigating the potential of poly(2-ethyl-2-oxazoline) and its polymer blends for enhancing Fenofibrate Amorphous Solid Dispersion Dissolution Profile

Ziru Zhang[1], Rasha ElKanayati[1], Sheng Feng[1], Indrajeet Karnik[1], Sateesh Kumar Vemula[1,2*], Michael A. Repka[1,3*]

[1] Department of Pharmaceutics and Drug Delivery, School of Pharmacy, The University of Mississippi, University, MS, 38677, USA.
[2] Department of Pharmaceutics, School of Pharmaceutical Sciences, Lovely Professional University, Phagwara, Punjab,144411, India.
[3] Pii Center for Pharmaceutical Technology, The University of Mississippi, University, MS, 38677, USA.
* Correspondence: svemula@olemiss.edu, marepka@olemiss.edu Tel.: +1 662 915 1155

Academic Editor: Firstname Lastname

Received: date
Revised: date
Accepted: date
Published: date

Citation: To be added by editorial staff during production.

Copyright: © 2025 by the authors. Submitted for possible open access publication under the terms and conditions of the Creative Commons Attribution (CC BY) license (https://creativecommons.org/licenses/by/4.0/).

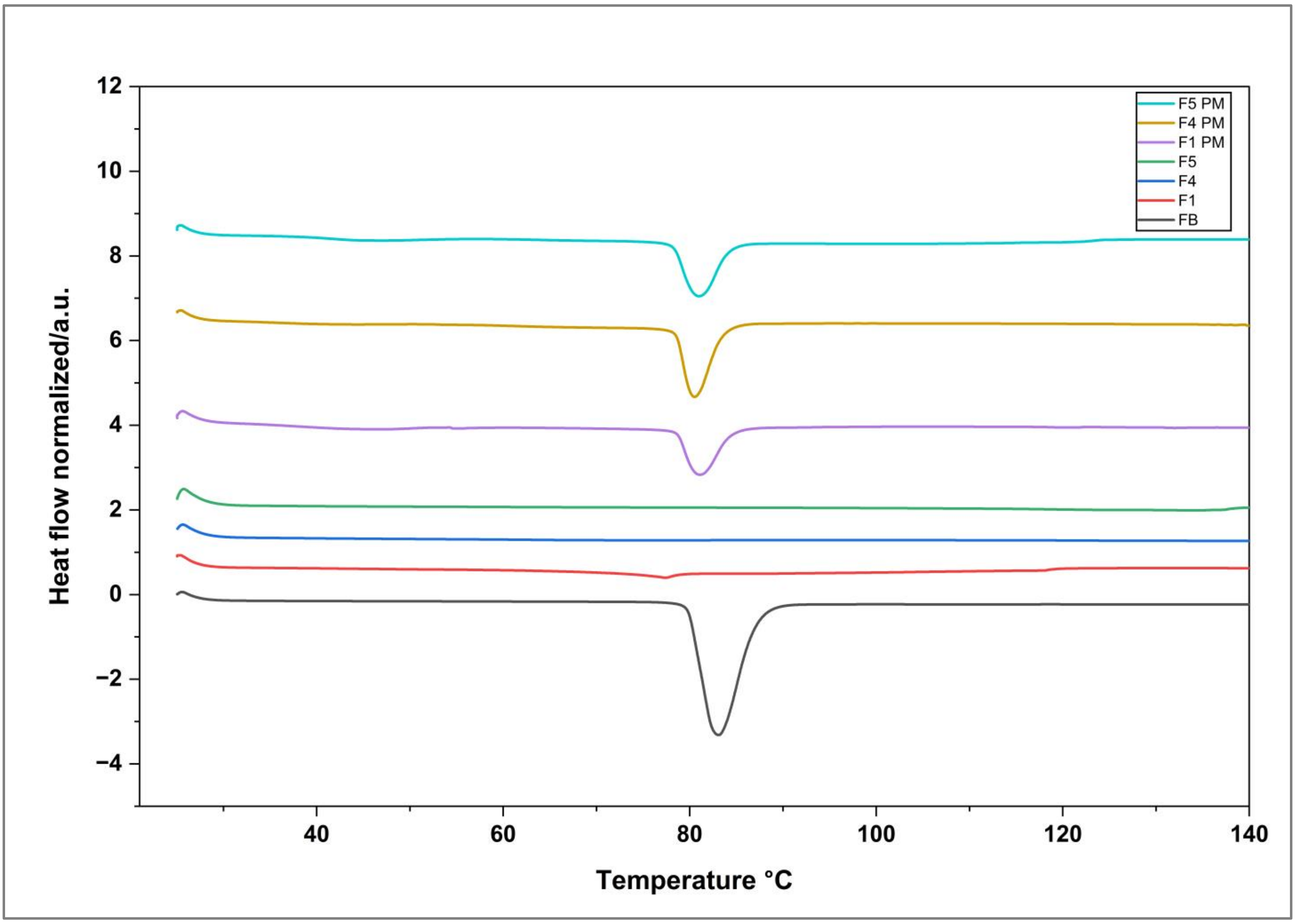

Figure S1. DSC of FB, selected ASDs and corresponding PMs

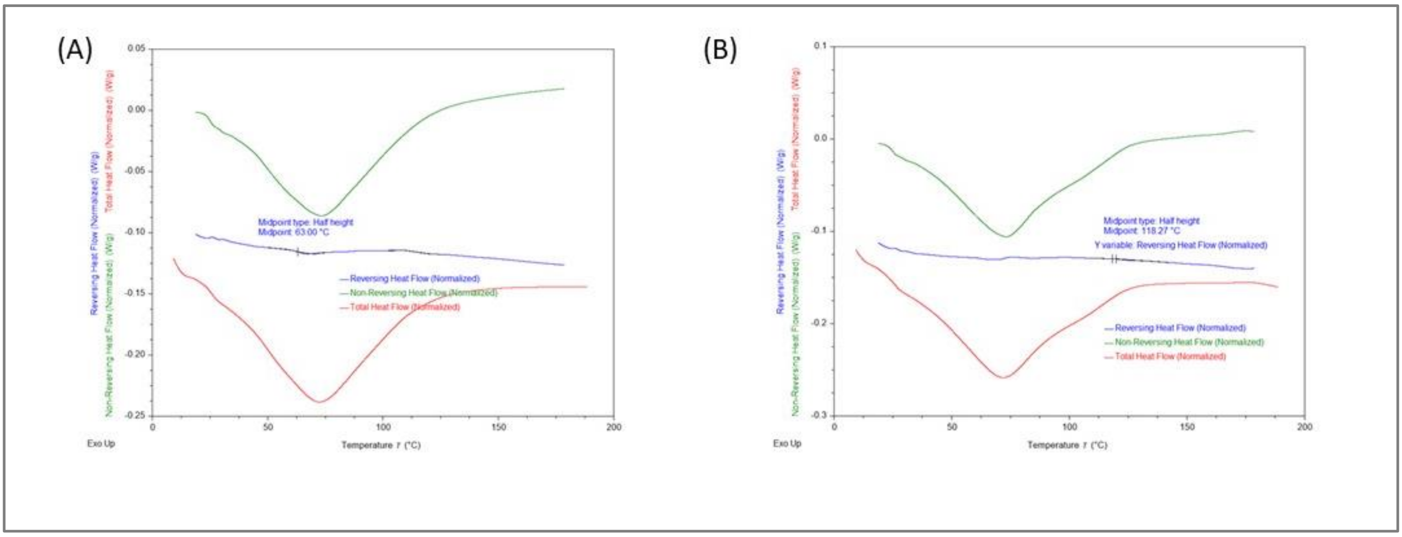

Figure S2. (A) M-DSC for F4 extrudate, and (B) F5 extrudate displaying a molecular dispersion with T_g of about 118 °C

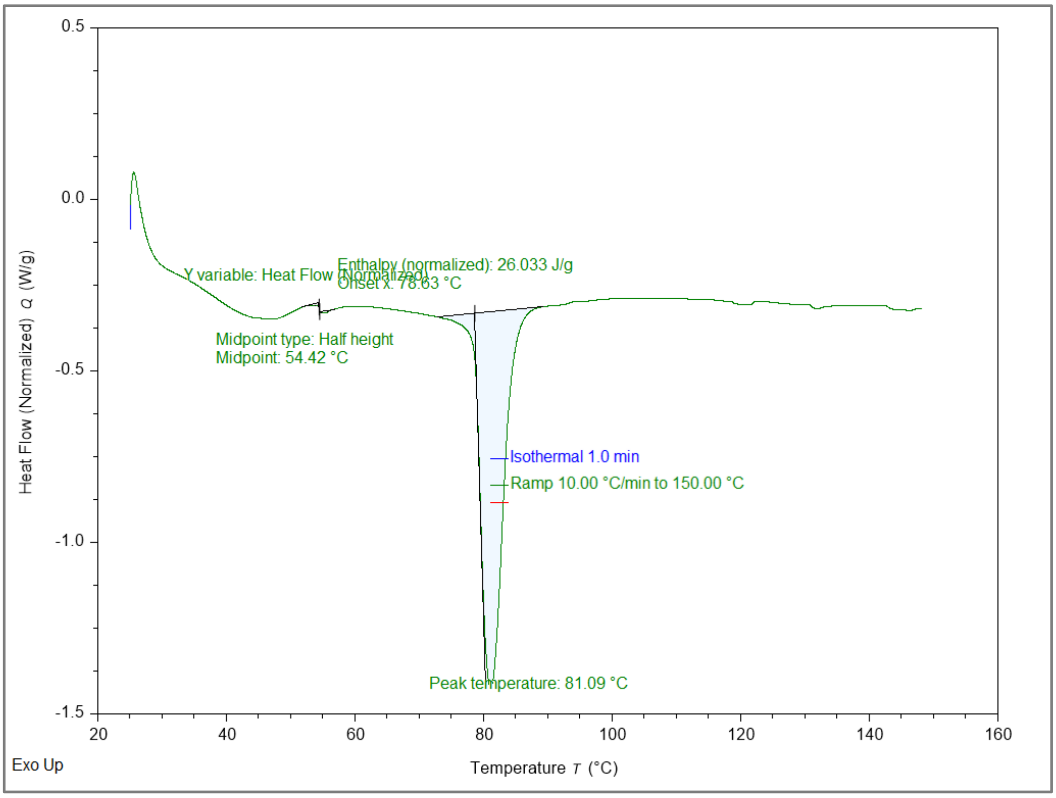

Figure S3. DSC of F1 PM demonstrating a melting endotherm of about 26 J/g

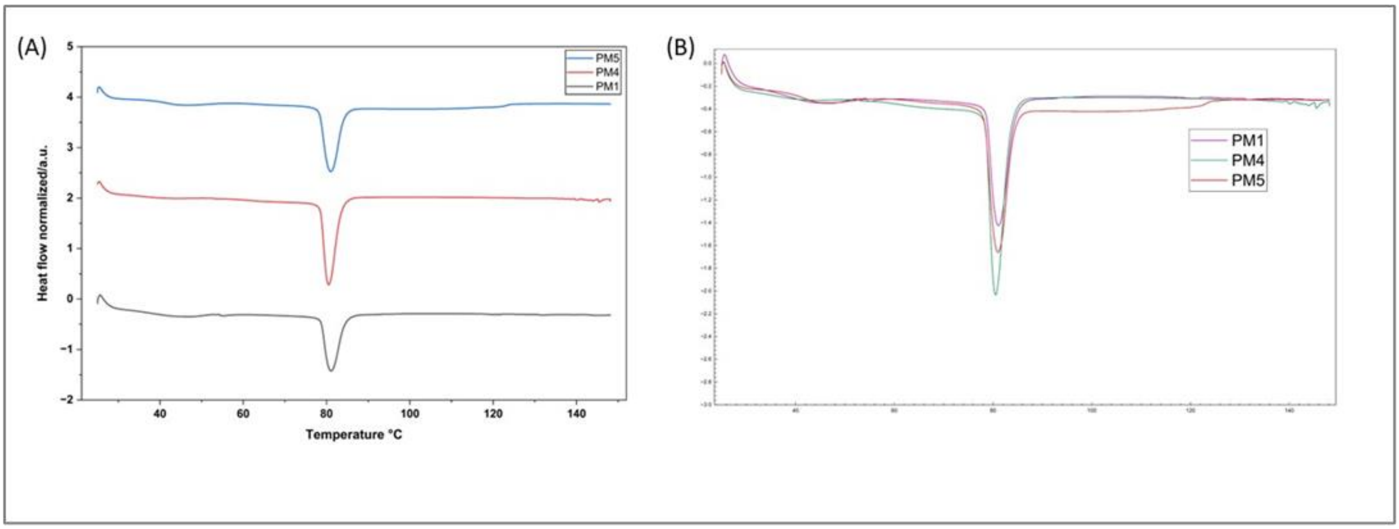

Figure S4. (A) DSC of PM1, PM4 and PM5 (B) Overlay of the physical mixtures showing lowest enthalpy of PM1 compared to PM4, and PM5
